# Peptidoglycan Hydrolases of Local Lactic Acid Bacteria from Kazakh Traditional Food

**DOI:** 10.5195/cajgh.2013.94

**Published:** 2014-01-24

**Authors:** Serik Shaikhin, Asel Moldagulova, Abzal Kazhybayev, Markhabat Kairova, Constantine Lee, Maira Urazova, Kairtai Almagambetov

**Affiliations:** Republican Collection of Microorganisms, Astana, Kazakhstan

**Keywords:** lactic acid, peptidoglycan hydrolases, multi-drug resistant strains, traditional food

## Abstract

**Introduction:**

Peptidoglycan (PG) is a major component of the cell wall of Gram-positive bacteria and is essential for maintaining the integrity of the bacterial cell and its shape. The bacteria synthesize PG hydrolases, which are capable of cleaving the covalent bonds of PG. They also play an important role in modeling PG, which is required for bacterial growth and division. In an era of increasing antibiotic-resistant pathogens, PG hydrolases that destroy these important structures of the cell wall act as a potential source of new antimicrobials. The aim of this study is to identify the main PG hydrolases of local lactic acid bacteria isolated from traditional foods that enhance probiotic activity of a biological preparation.

**Methods:**

*Lactococcus lactis* 17A and *Lactococcus garvieae* 19A were isolated from the traditional sausage-like meat product called kazy. They were isolated according to standards methods of microbiology. Genetic identification of the isolates were tested by determining the nucleotide sequences of 16S rDNA. The Republican collection of microorganisms took strains of *Lactobacillus casei subsp. Rhamnosus* 13-P, *L. delbrueckii subsp*. *lactis* CG-1 B-RKM 0044 from cheese, *Lactobacillus casei subsp. casei* B-RKM 0202 from homemade butter. They used the standard technique of renaturating polyacrylamide gel electrophoresis to detect PG hydrolases activity.

**Results:**

According to the profiles of PG hydrolase activity on zymograms, the enzymes of Lactococci 17A and 19A in kazy are similar in electrophoretic mobility to major autolysin AcmA, while the lactobacilli of industrial and home-made dairy products have enzymes similar to extracellular proteins p40 and p75, which have probiotic activity.

**Conclusions:**

Use of peptidoglycan hydrolases seems to be an interesting approach in the fight against multi-drug resistant strains of bacteria and could be a valuable tool for the treatment of diseases caused by these microorganisms in Kazakhstan.

